# Synthesis of Keggin-Al_13_-modified bentonite materials with enhanced mesoporosity: structural and textural characterization and application in soot oxidation

**DOI:** 10.1039/d6ra07334d

**Published:** 2026-11-10

**Authors:** Samra Benkacem, Walid Boultif, Youghourta Belhocine, Djihane Slimane Ben Ali

**Affiliations:** a Laboratory of Chemical Engineering and Environment of Skikda - LGCES, 20 August 1955, University of Skikda P.O. Box 26, El Hadaik Road Skikda 21000 Algeria; b Laboratory of Catalysis, Bioprocess and Environment, 20 August 1955, University of Skikda, x 26, El H P.O. Bo adaik Road Skikda 21000 Algeria; c Department of Petrochemical, Faculty of Technology, University of 20 Août1955 El Hadaiek Road, B. O. 26 21000 Skikda Algeria s.benkacem@univ-skikda.dz

## Abstract

This paper reports the preparation of a series of bentonite-based materials through sequential sodium exchange, sulfuric acid activation, Al_13_ polycation treatment, and subsequent cobalt (Co) and manganese (Mn) impregnation. The structural and textural properties of the resulting materials were investigated using FTIR, XRD, TEM, TGA–DTA, BET surface area analysis, and fractal analysis. The soot oxidation performance was measured using O_2_ with *T*_10_, *T*_50_ and *T*_90_ as conversion indicators. Acid activation markedly modified the structure and texture of the bentonite, resulting in a significant increase in specific surface area, reaching 438.84 m^2^ g^−1^ for BNT-NaA, together with the development of mesoporosity. XRD analysis confirmed the expansion of the basal spacing after Al_13_ treatment of raw and sodium-exchanged bentonite, with *d*_001_ values of 16.96 and 18.03 Å for BNT-P and BNT-NaP, respectively, indicating interlayer incorporation of Al_13_ polycations. In contrast, the acid-activated samples subsequently treated with Al_13_, BNT-AP and BNT-NaAP, did not exhibit a well-defined *d*_001_ reflection, indicating substantial disruption of the original layered structure and preventing the confirmation of a well-ordered pillared framework by XRD. Nevertheless, BNT-NaAP retained a relatively high specific surface area of 210.42 m^2^ g^−1^ and pronounced mesoporous characteristics. Following metal impregnation, Co/BNT-NaAP and Mn/BNT-NaAP exhibited specific surface areas of 219.75 and 187.92 m^2^ g^−1^, respectively. Fractal analysis revealed an increase in surface heterogeneity following the modification treatments, with fractal dimension values ranging from 2.61 to 2.95. Co/BNT-NaAP had a *T*_50_ of 510 °C, compared to 590 °C for BNT-NaAP and 660 °C for soot alone, indicating enhanced catalytic activity under the investigated conditions.

## Abstract 2

This paper reports the preparation of a series of bentonite-based materials through sequential sodium exchange, sulfuric acid activation, Al_13_ polycation treatment, and subsequent cobalt (Co) and manganese (Mn) impregnation.

## Graphical abstract

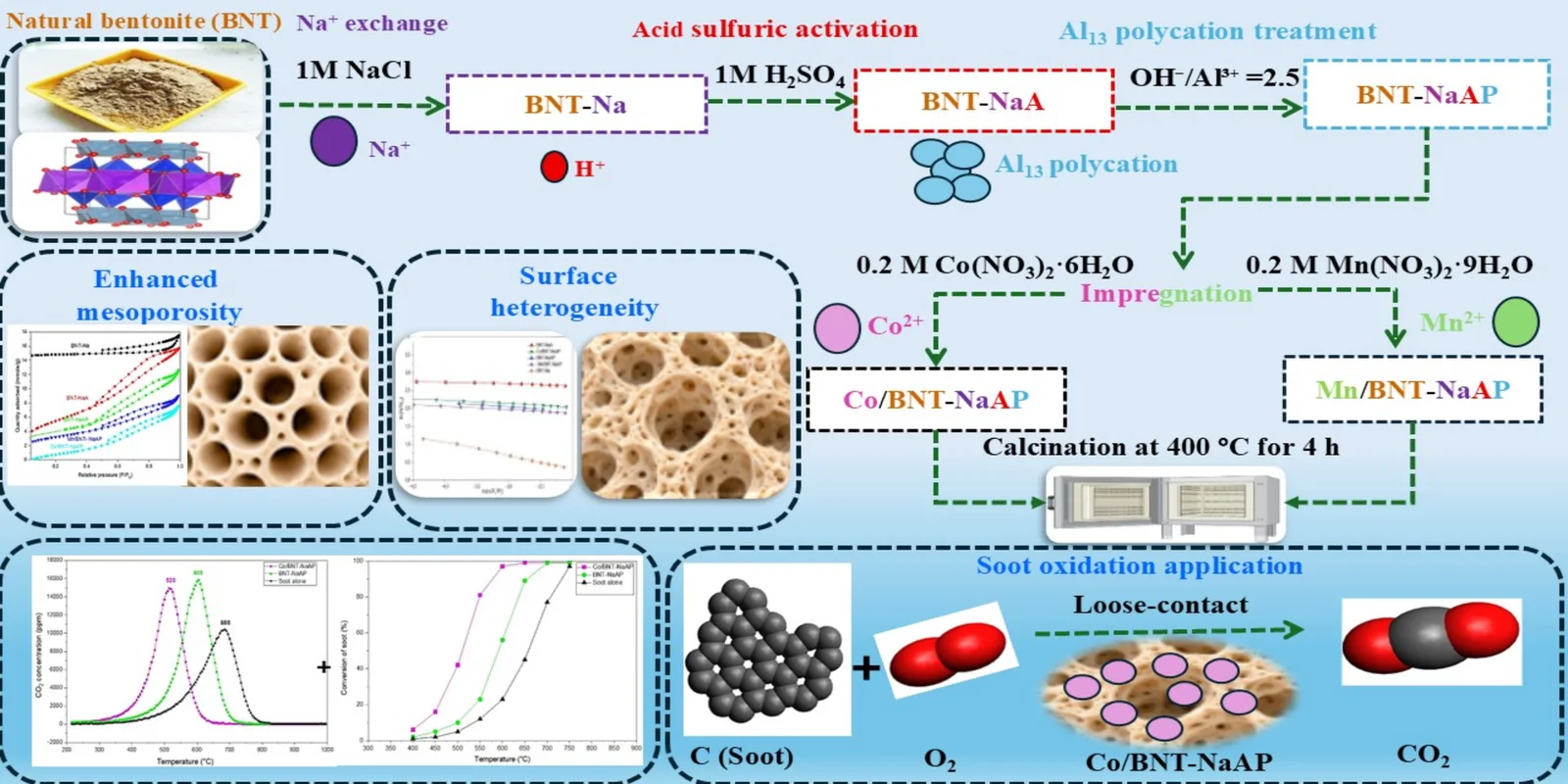

## 1. Introduction

Clays are naturally occurring aluminosilicate minerals composed predominantly of fine-grained particles and are typically rich in elements such as silicon, aluminum, iron, and magnesium.^1^ Clays have been widely studied for their various applications such as environmental remediation,^2–4^ adsorption,^5–7^ catalysis^8–10^ and the ceramics industry^11,12^ due to their abundance, low cost and their unique physicochemical properties. An especially important application of them is as catalytic supports.^13^ In heterogeneous catalysis, the support materials offer stable platforms for dispersion of active catalytic species, thus improving their catalytic efficiency and thermal stability.^14^ Clays are particularly well suited to this role thanks to their high specific surface area, layered structure, ion-exchange capacity, thermal resistance and tunable surface chemistry.^15^

Clay minerals such as bentonite,^16^ kaolinite,^17^ illite,^18^ palygorskite^19^ and sepiolite^20^ have been widely used as catalyst supports. These materials can be used in natural form or can be further modified by acid activation, thermal treatment, pillaring or impregnation with metal salts^21^ to improve their physicochemical properties. These changes may significantly improve the pore architecture, surface acidity and mechanical stability of the clay, consequently improving the efficiency, selectivity and durability of the catalytic system.^14^

Clay-supported catalysts have found applications in diverse catalytic processes, including fluid catalytic cracking (FCC),^22,23^ hydrodesulfurization,^24^ decomposition of volatile organic compounds (VOCs),^25^ wastewater treatment,^26^ biodiesel production,^27^ and selective oxidation reactions.^28^ Thus, clay catalytic supports are a cheap and sustainable alternative to traditional inorganic materials, in line with the principles of green chemistry and the circular economy.

Bentonite, a member of the smectite clay group, is one of the most studied clay minerals in catalytic applications.^29^ It is a 2 : 1 structure with a central octahedral sheet of alumina between two tetrahedral sheets of silica. This architecture allows the intercalation of bentonite with a large variety of organic and inorganic species and thus enables it to be a highly versatile support material. The ability to host organic molecules makes bentonite a suitable host for organocatalysts.^30^

In addition, bentonite has several beneficial textural and physicochemical properties such as high specific surface area, large pore volume, well-ordered lamellar structure and excellent cation exchange capacity. These properties, along with its mechanical robustness and chemical stability, make bentonite a very suitable material to be used as a catalyst and an adsorbent in a variety of chemical processing industries.^31^

The pillaring process is achieved through a cation exchange mechanism, in which strong inorganic polycations are introduced into the interlayer spaces of clay minerals.^32^ Subsequent thermal treatment decomposes these intercalated species to form metal oxide pillars which are tightly bound between clay layers to stabilize and expand the interlayer structure. Hydroxyl polycations of polynuclear metals such as aluminum (Al), iron (Fe), zirconium (Zr), chromium (Cr), and titanium (Ti) are among the most extensively studied inorganic pillaring agents.^22^ These species not only increase the thermal and mechanical stability of the resulting materials, but also increase the surface area and porosity, making pillared clays attractive for catalytic and adsorption applications.^33^

Hao *et al.* investigated a bifunctional Co/pillared clay catalyst system and demonstrated that pillaring montmorillonite with SiO_2_, Al_2_O_3_, and ZrO_2_ significantly enhanced both basal spacing and surface area compared to untreated and acid-activated clays.^34^ Notably, Al- and Si-pillared montmorillonites exhibited pronounced increases in interlayer spacing. These materials also showed marked improvements in BET surface area; approximately 75% of the total BET surface area was associated with micropores. Pore size distribution analysis revealed that the pillared interlayered clays (PILCs) possess hierarchical porosity with a well-balanced distribution of micro- and mesopores, which is advantageous for diffusion-limited catalytic processes. The enhanced dispersion of cobalt and improved catalytic activity in Fischer–Tropsch synthesis was directly linked to the structural modifications imparted by the pillaring process.

In related work, bentonite clays pillared with mixed aluminum–cobalt polycations have been synthesized and characterized by Bertella *et al.*^35^ When the cobalt content was varied from 10% to 100%, effective pillaring was observed for Co concentrations of 10 and 25% with expanded basal spacings up to 19.3 Å and high BET surface areas (up to 334 m^2^ g^−1^) which are higher than those of clays pillared with aluminum alone. However, at Co contents above 50% true pillared structures were not observed but the formation of layered double hydroxides (LDHs) or discrete cobalt oxides were formed. These results suggest that mixed Al/Co polycations at optimized ratios (≤25% Co) may significantly improve the textural properties of bentonite for catalytic applications.

Soot oxidation is an important process for controlling particulate emission from diesel exhaust gases. Catalysts can promote the oxidation rate of soot, but the efficiency strongly depends on the contact between the soot particles and the catalytic material. Neeft *et al.*^36^ demonstrated that a large number of catalysts are significantly more active under tight-contact conditions than under loose-contact conditions. These results show the importance of considering the contact between soot and catalyst in the evaluation of materials for this application.

The primary objective of the first part of this study was to structurally modify bentonite clay to make it more suitable as a catalytic support. This was obtained by successive cation exchange and acid activation with H_2_SO_4_ followed by intercalation of Al_13_ polycations (Keggin-type ions)^37^ in the interlamellar spaces. The objective of this was to maximize the spacing between the layers and so to encourage the formation of a stable porous framework.

From an economic point of view, clay-based catalysts are recognized increasingly as economically viable alternatives to conventional catalytic materials. The results of this work underline the promising potential of these modified clays as a new generation of catalytic supports, which can be further functionalized by impregnation with metal precursor salts to give active and efficient catalysts.

The second objective was to investigate the possible use of the modified bentonite as a support for cobalt-containing catalysts for the soot oxidation under O_2_. The experimental evaluation of this application requires the comparison of BNT-NaAP, Co/BNT-NaAP and soot alone based on CO_2_ evolution profiles and characteristic conversion temperatures *T*_10_, *T*_50_ and *T*_90_.

## 2. Materials and methods

### 2.1. Materials and equipment

The raw material used in this study was natural industrial grade bentonite from Tlemcen, Algeria, which is a well-known area of high purity smectite-rich clay deposits. The bentonite has a general chemical formula (Na,Ca)_0.33_(Al,Mg)_2_Si_4_O_10_(OH)_2_·*n*H_2_O and is generally characterized by a light gray to beige color, fine-grained texture and soft consistency. The particle size of the as-received material was small enough (<60 µm) that no additional grinding was required. The bentonite was air-dried and then oven-dried at 110 °C to remove residual moisture. For simplicity, this will be referred to as BNT.

The absolute density of the raw bentonite (BNT) measured by a helium pycnometer was 2.70 g cm^−3^. The material was dry milled in a Retsch PM100 ball mill for 15 minutes for homogeneity. The elemental composition of BNT was obtained by X-ray fluorescence (XRF) spectroscopy with a Rigaku ZSX Primus IV instrument (see Table S1). The results revealed silica (SiO_2_) as the major oxide followed by alumina (Al_2_O_3_) which is a typical mineralogical phase in the smectite-rich clays.

To enable accurate characterization of the phyllosilicate structure, it was necessary to isolate the clay fraction, defined as particles smaller than 2 µm. This separation was carried out using the classical method based on differential sedimentation, in accordance with Stokes' law, which allows the separation of fine clay particles from denser and coarser mineral impurities.^38^ Furthermore, a cation exchange procedure was used to convert the sample into its homoionic form for further structural and textural changes.

Fourier-transform infrared (FTIR) spectra of the clay samples were recorded in the range of 4000–400 cm^−1^ using KBr pellet methodology on an ATI Mattson spectrometer. X-ray diffraction (XRD) patterns of the raw and modified bentonite (BNT) samples were obtained using an X'Pert PRO diffractometer (PANalytical) equipped with a graphite-monochromated Cu K_α_ radiation source (*λ* = 1.5406 Å). The morphology and microstructure of the materials were studied by transmission electron microscopy (TEM) on Topcom EM-002B microscope. Thermogravimetric analysis (TGA) and differential thermal analysis (DTA) were performed on a NETZSCH STA 449 F3 Jupiter thermal analyser in air from room temperature to 750 °C with a heating rate of 10 °C min^−1^.

Nitrogen adsorption–desorption isotherms were measured at 77 K using an ASAP Micromeritics system. Prior to analysis, all samples were degassed under vacuum at 250 °C overnight to remove physisorbed moisture and volatile contaminants from the surface. The specific surface area (*S*_BET_) was calculated using the Brunauer–Emmett–Teller (BET) method, applied to the adsorption data within the relative pressure (*P*/*P*_0_) range of 0.05–0.30. Pore size distributions were determined from both the adsorption and desorption branches of the isotherms using the Barrett–Joyner–Halenda (BJH) method.^39^

The surface fractal dimension (*D*) was estimated using the N_2_ adsorption isotherms measured at 77 K based on the Frenkel–Halsey–Hill (FHH) model.^40^ In the capillary-condensation formulation the model is given as:1
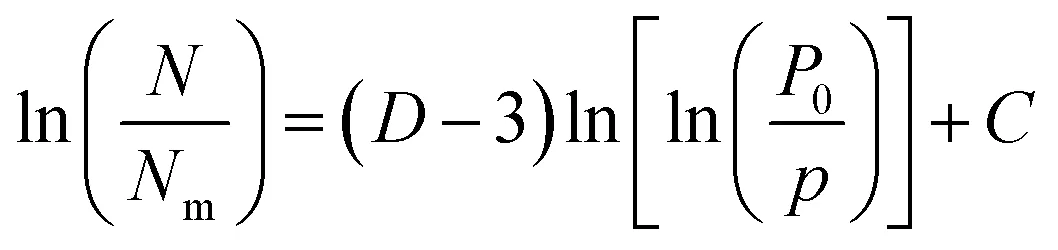
where *N* is the amount of nitrogen adsorbed at the equilibrium pressure *p*, *N*_m_ is the monolayer adsorption capacity, *P*_0_ is the saturation vapour pressure of nitrogen and *C* is a constant. The fractal dimension was computed as *D* = 3 + *A* where *A* is the slope of the linear fit of 
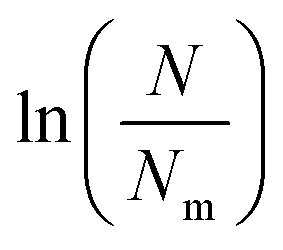
*versus*
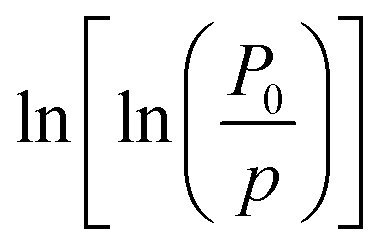
. Values near 2 indicate relatively smooth surfaces while values near 3 indicate more surface roughness and geometrical irregularity.

All chemicals and reagents used in this study were of analytical grade. These included sodium chloride (NaCl, 99.0%), sulfuric acid (H_2_SO_4_, 96%), and hydrogen peroxide (H_2_O_2_, 30% w/w), all purchased from Sigma-Aldrich. The precursor salts used for modification were aluminum nitrate nonahydrate (Al(NO_3_)_3_·9H_2_O), cobalt nitrate hexahydrate (Co(NO_3_)_2_·6H_2_O), and manganese nitrate nonahydrate (Mn(NO_3_)_2_·9H_2_O), all of which were also of analytical grade.

### 2.2. Preparation of samples

A total of 30 g of crushed raw bentonite (BNT) was dispersed in H_2_O_2_ and magnetically stirred at room temperature for 2 h to remove organic matter. The suspension was carefully washed and treated with three successive cation-exchange treatments with 1 M NaCl to obtain sodium-exchanged bentonite (BNT-Na). The solid was collected by centrifugation after each treatment and was washed several times with distilled water to remove residual chloride ions. Absence of chloride ions was confirmed by AgNO_3_. The resulting BNT-Na was dried at 80 °C.

20 g of BNT-Na were taken in a round bottom flask and treated with 1 L of 1 M H_2_SO_4_ solution for acid activation. The suspension was heated at about 98 °C and maintained under reflux with continuous stirring for 6 h. After treatment, the solid was recovered by centrifugation and repeatedly washed with distilled water until sulfate ions were no longer detected in the filtrate. The acid-activated material was dried at 80 °C and designated BNT-A.

Prior to pillaring, the clay samples were dried and finely ground. The Al_13_ pillaring solution was prepared by controlled hydrolysis of a 0.2 M aqueous Al (NO_3_)_3_ solution with NaOH. The NaOH solution was added dropwise under continuous stirring until an OH^−^/Al^3+^ molar ratio of 2.5 was reached. The resulting solution was aged for 24 h at room temperature to promote the formation of Keggin-type Al_13_ polycations. The freshly prepared pillaring solution was then separately added to aqueous suspensions of BNT, BNT-Na, BNT-A, and BNT-NaA at a nominal Al loading of 10 mmol Al per g clay. The suspensions were continuously stirred at room temperature for 24 h to promote the intercalation of Al_13_ polycations into the interlayer spaces. The solids were subsequently recovered by centrifugation, thoroughly washed with distilled water, and dried at 50 °C.^41^ The resulting materials were designated BNT-P, BNT-NaP, BNT-AP, and BNT-NaAP, respectively.

Cobalt- and manganese-containing catalysts were subsequently prepared by the wet impregnation method using BNT-NaAP as the support. Cobalt nitrate hexahydrate (Co(NO_3_)_2_·6H_2_O) and manganese nitrate nonahydrate (Mn(NO_3_)_2_·9H_2_O) were used as the cobalt and manganese precursors, respectively. Aqueous 0.2 M solutions of Co(NO_3_)_2_·6H_2_O and Mn(NO_3_)_2_·9H_2_O were prepared and used for the impregnation of BNT-NaAP. The impregnation was performed using a fixed liquid-to-solid mass ratio of 14 : 86, with identical precursor concentrations for both metals. The resulting suspensions were continuously stirred to promote the homogeneous distribution of the metal species over the clay support. After impregnation, the samples were dried and finely ground, followed by calcination in a muffle furnace under air at 400 °C for 4 h. The resulting materials were designated Co/BNT-NaAP and Mn/BNT-NaAP, respectively.

#### 2.2.1. Sample designation

The prepared samples were designated according to the treatment applied: BNT (raw bentonite), BNT-Na (sodium-exchanged bentonite), BNT-A (acid-activated bentonite), BNT-P (Al_13_-pillared raw bentonite), BNT-AP (acid-activated and Al_13_-pillared bentonite), BNT-NaA (sodium-exchanged and acid-activated bentonite), BNT-NaP (sodium-exchanged and Al_13_-pillared bentonite), BNT-NaAP (sodium-exchanged, acid-activated, and Al_13_-pillared bentonite), and Co/BNT-NaAP and Mn/BNT-NaAP (Co- and Mn-impregnated BNT-NaAP, respectively).

### 2.3. H_2_ temperature-programmed reduction

Hydrogen temperature-programmed reduction (H_2_-TPR) was used to study the reduction behaviour of the supports and cobalt-containing catalyst with the experimental setup shown in Fig. S1. A quartz reactor in a programmable furnace was loaded with 50 mg of sample and heated at 15 °C min^−1^ to 900 °C in flowing 10% H_2_/Ar for each measurement. Sample temperature was monitored by thermocouples and changes in H_2_ concentration were monitored by a thermal conductivity detector (TCD). The H_2_-TPR profiles were recorded as the detector signal against temperature.

### 2.4. Catalytic soot oxidation tests

Catalytic soot oxidation was conducted by a method adapted from Gebretsadik *et al.*^42^ The experiments were performed in a tubular quartz reactor with continuous gas analysis. Model soot was Printex U carbon black (Evonik-Degussa). In each catalytic test, 20 mg soot was mixed with 80 mg catalyst under loose-contact condition, which corresponds to a soot to catalyst mass ratio of 1 : 4. To improve heat transfer and reduce pressure drop, the mixture was diluted with SiC. The temperature was increased to 800 °C at a heating rate of 10 °C min^−1^, in a mixture of 10 vol% O_2_ balanced with N_2_, with no addition of NO, and a total flow rate of 500 mL min^−1^. Continuous measurements of CO_2_ concentrations were made.

The catalytic activities of BNT-NaAP and Co/BNT-NaAP were tested under the same experimental conditions and soot only was tested as an uncatalyzed reference. Neglecting CO formation, the conversion of soot was calculated from the cumulative carbon released as CO_2_ after background correction relative to the initial carbon content of soot. The catalytic performance was assessed as *T*_10_, *T*_50_ and *T*_90_ which means the temperature for 10%, 50% and 90% soot conversion, respectively. The maximum CO_2_ evolution temperature was designated as *T*_m_.

## 3. Results and discussion

### 3.1. FTIR analysis

FTIR spectra of the non-calcined bentonite-based samples and the band assignments are presented in Fig. 1 and 2. The spectra show the characteristic vibrational features of the bentonite aluminosilicate framework. The absorption bands observed at approximately 3698 and 3620 cm^−1^ are assigned to the O–H stretching vibrations of structural hydroxyl groups associated with the clay layers. The broad band centered around 3450 cm^−1^ is mainly attributed to the O–H stretching vibrations of adsorbed and interlayer water molecules.^43^

**Fig. 1:**
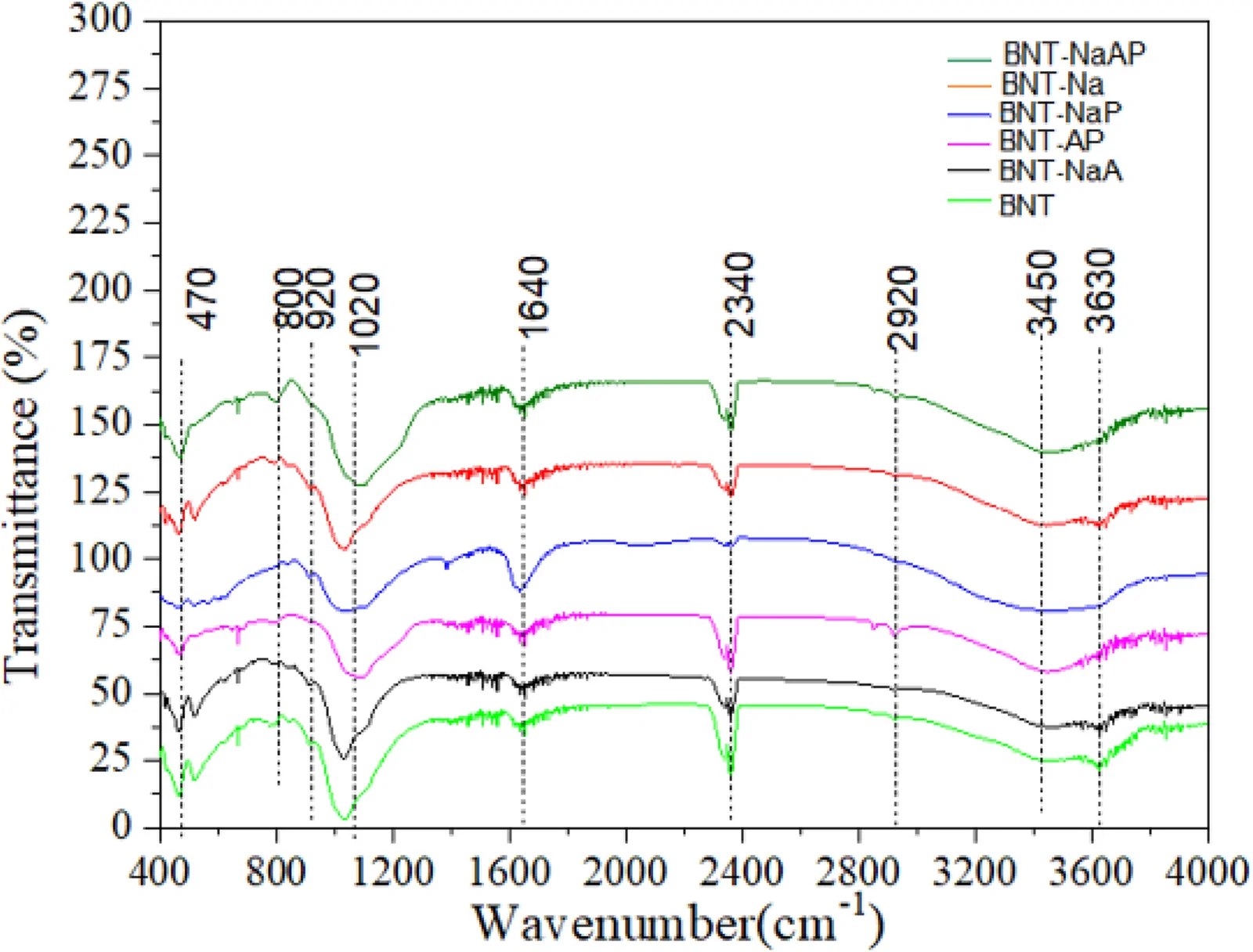
FTIR spectra of non-calcined samples BNT, BNT-Na, BNT-NaA, BNT-AP, BNT-NaP and BNT-NaAP.

**Fig. 2:**
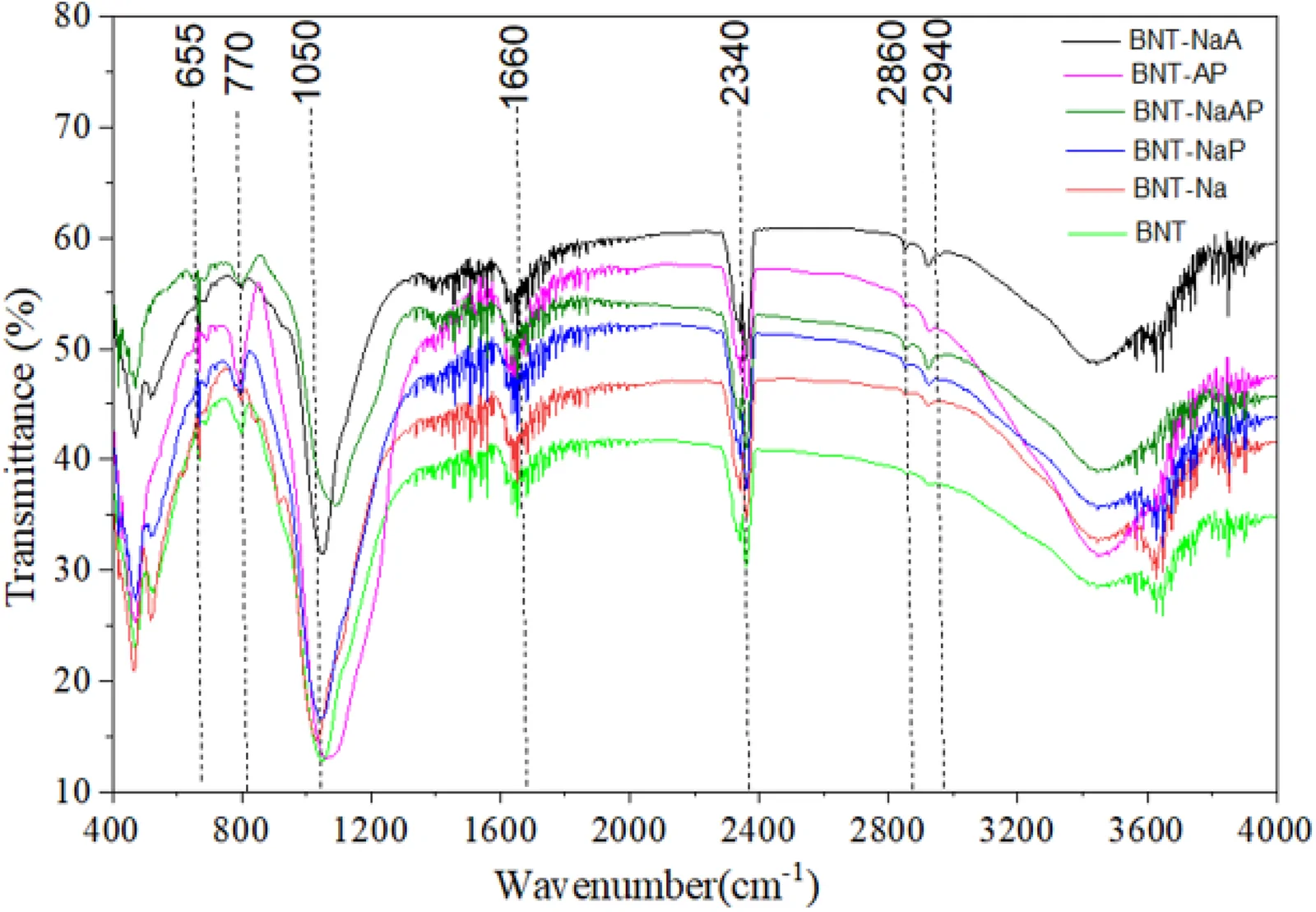
FTIR spectra of calcined bentonite samples (400 °C): BNT, BNT-Na, BNT-NaA, BNT-AP, BNT-NaP and BNT-NaAP.

The band observed in the region of 1630–1650 cm^−1^ is assigned to the H–O–H bending vibration of adsorbed water.^44^ A weak band around 2340 cm^−1^ can be attributed to atmospheric CO_2_. The intense absorption band in the 1000–1100 cm^−1^ region is characteristic of Si–O stretching vibrations of the aluminosilicate framework. The bands observed at lower wavenumbers are associated with vibrations involving Si–O–Al and other metal–oxygen linkages characteristic of layered aluminosilicates.^45^

In contrast to raw bentonite (BNT), the sodium-exchanged sample (BNT-Na) maintains the primary FTIR bands linked to the aluminosilicate structure, indicating that this structure is largely preserved during sodium exchange. Fluctuations in band intensity and morphology may indicate alterations in the interlayer chemical milieu subsequent to cation exchange.^46^ Supplementary bands may emerge from minor mineral constituents; yet, overlapping vibrations hinder their definitive identification using FTIR alone. Consequently, the allocation of accessory phases like quartz or calcite must be evaluated in conjunction with the XRD findings.^47,48^

After acid activation, the FTIR spectrum of BNT-NaA shows significant changes in the O–H stretching and Si–O stretching regions. The intensity of the O–H stretching band decreases because of changes in the hydroxyl environment and partial alteration of the octahedral sheet during the acid treatment.^49^ Alterations in the Si–O region are in agreement with modifications of the aluminosilicate framework due to acid activation. However, FTIR spectroscopy alone cannot quantify the dealumination extent or directly quantify the leaching of Fe, Al and Mg.

The comparison of the BNT-Na and BNT-NaP spectra demonstrates the intensity and profile change of the O–H and H–O–H vibration bands after treatment with the Al_13_ containing species. These changes suggest changes in the interlayer chemical environment and interactions between clay layers and Al-containing species. The observed changes in the spectra are consistent with the incorporation of Al-containing species during the pillaring treatment. However, FTIR alone cannot conclusively establish the formation of an ordered Al_13_-pillared structure; this interpretation should therefore be considered together with the XRD and textural characterization results.

The FTIR spectra of the modified samples before and after calcination at 400 °C (see Fig. 1 and 2) show further changes, particularly in the O–H stretching and H–O–H bending regions. These changes are mainly associated with the removal of adsorbed/interlayer water and thermal modification of structural hydroxyl groups.

The changes observed in the 800–900 cm^−1^ region could be related to the alteration of the aluminosilicate framework caused by the thermal treatment. However, FTIR bands in clay minerals may overlap and correspond to different structural environments, so these changes cannot be uniquely assigned to the formation of amorphous silica or structural degradation based on FTIR alone. Therefore, additional characterization techniques, especially XRD, are required for more conclusive interpretation.^50,51^

In general, FTIR results indicate that the sodium exchange, acid activation, Al_13_ treatment and calcination change the chemical environment of the bentonite, but the main characteristic vibrations of the aluminosilicate framework are maintained. The FTIR results should be interpreted together with the XRD and BET results to get a complete picture of the structural and textural changes induced by the different treatments.

### 3.2. X-ray diffraction analysis

The X-ray diffraction (XRD) pattern of the raw bentonite sample (BNT) is shown in Fig. 3 and shows the characteristic reflections of bentonite, the predominant mineralogical phase. The basal reflection corresponding to the (001) plane is observed at 2*θ* = 6.68°, corresponding to an interlayer spacing (*d*_001_) of 13.23 Å.^52^ In addition, a sharp and intense reflection at 2*θ* = 20.08° (*d*_110_ = 4.45 Å) is assigned to the (110) plane, whereas the broader reflection centered at approximately 2*θ* = 35° (*d*_200_ = 2.65 Å) corresponds to the (200) plane of the bentonite structure. Minor crystalline phases, such as feldspar (*d* ≈ 2.50 Å), quartz (*d* ≈ 3.33 Å) and calcite (*d* ≈ 4.22 Å) indicating the presence of accessory mineral phases in the natural bentonite.

**Fig. 3:**
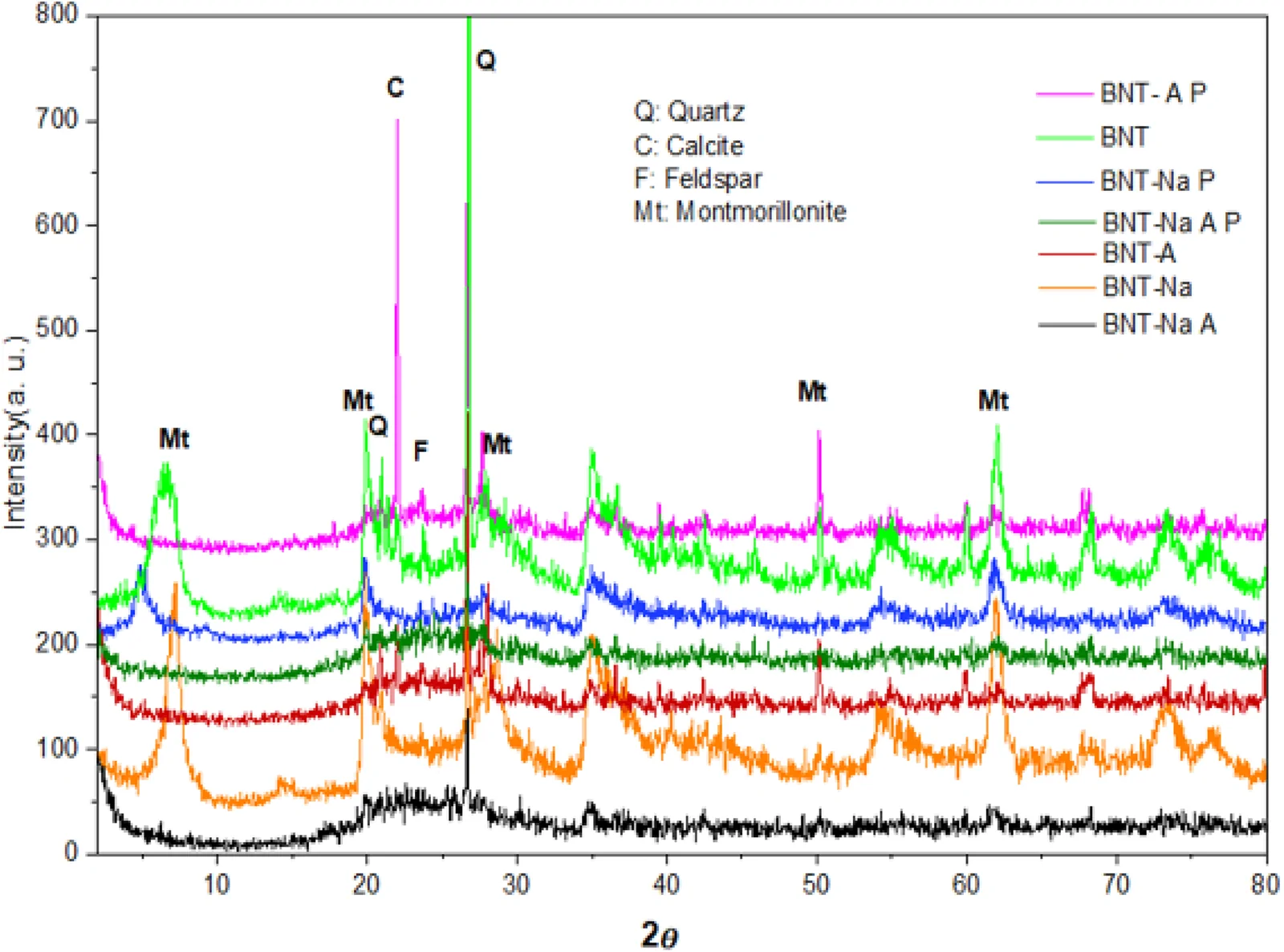
X-ray diffraction (XRD) patterns of raw bentonite and its chemically modified derivatives.

The shift of the basal (001) reflection to a higher angle of diffraction relative to the raw bentonite is shown in the BNT-Na sample (see Fig. 3), which indicates a decrease in the basal spacing. Such behavior implies a change in the interlayer environment upon Na^+^ exchange and suggests removal or substitution of the original interlayer cations and associated species.^53^

The XRD pattern of the acid activated bentonite (BNT-NaA) presents significant changes on the crystalline structure. In particular the loss of the basal reflection (001) suggests a strong loss of long-range order in the montmorillonite layers. The structural transformation is explained by the action of sulfuric acid, which favors the leaching of structural cations, particularly Al and Mg, from the octahedral sheet. This structural degradation and partial loss of layered organization result in the absence of a clearly detectable *d*_001_ reflection. The relative intensity of the quartz reflection at 2*θ* ≈ 26.70° (*d* ≈ 3.34 Å) is increased mainly due to the preferential dissolution of the clay fraction during acid activation, which makes the quartz phase relatively more prominent in the diffractogram.^54^

The XRD patterns of BNT-P and BNT-NaP show strong basal reflections at 2*θ* = 5.21° and 4.90° with *d*_001_ spacings of 16.96 and 18.03 Å respectively. The shift of the basal reflection to lower diffraction angles along with the increase of basal spacing in comparison with raw bentonite indicates an expansion of the interlayer region after treatment with the Al_13_ polycation. The results suggest that the Al_13_ species intercalate between the clay layers. The larger basal spacing of BNT-NaP suggests that the previous Na^+^ exchange process promoted the interlayer incorporation of the Al_13_ polycations. Therefore, the XRD results indicate that BNT-P and BNT-NaP have structural characteristics similar to Al_13_-pillared bentonite materials. The enhanced basal spacings obtained for BNT-P and BNT-NaP thus agree with those reported for Al-pillared bentonite materials.^55^

In contrast, the XRD patterns of the acid-activated samples treated with Al_13_ polycations, BNT-AP and BNT-NaAP, do not present a well-defined basal (001) reflection. This behavior shows that the treatment with sulfuric acid led to a significant destruction of the original layered structure. The lack of a *d*_001_ reflection after the subsequent Al_13_ treatment indicates that the original lamellar order was not recovered. For BNT-AP and BNT-NaAP, the lack of a well-defined basal reflection prevents confirmation of a well-ordered Al_13_-derived pillared structure by XRD alone. This does not prove the absence of pillars; complementary characterization would be necessary to determine the nature and the organization of the incorporated Al-containing species.

The XRD pattern of the calcined bentonite at 400 °C (see Fig. 4) reveals a significant decrease in the intensity of the basal (001) reflection of the bentonite, at about 2*θ* ≈ 6.5°. This decrease agrees with the loss of interlayer water and structural changes caused by thermal treatment.

**Fig. 4:**
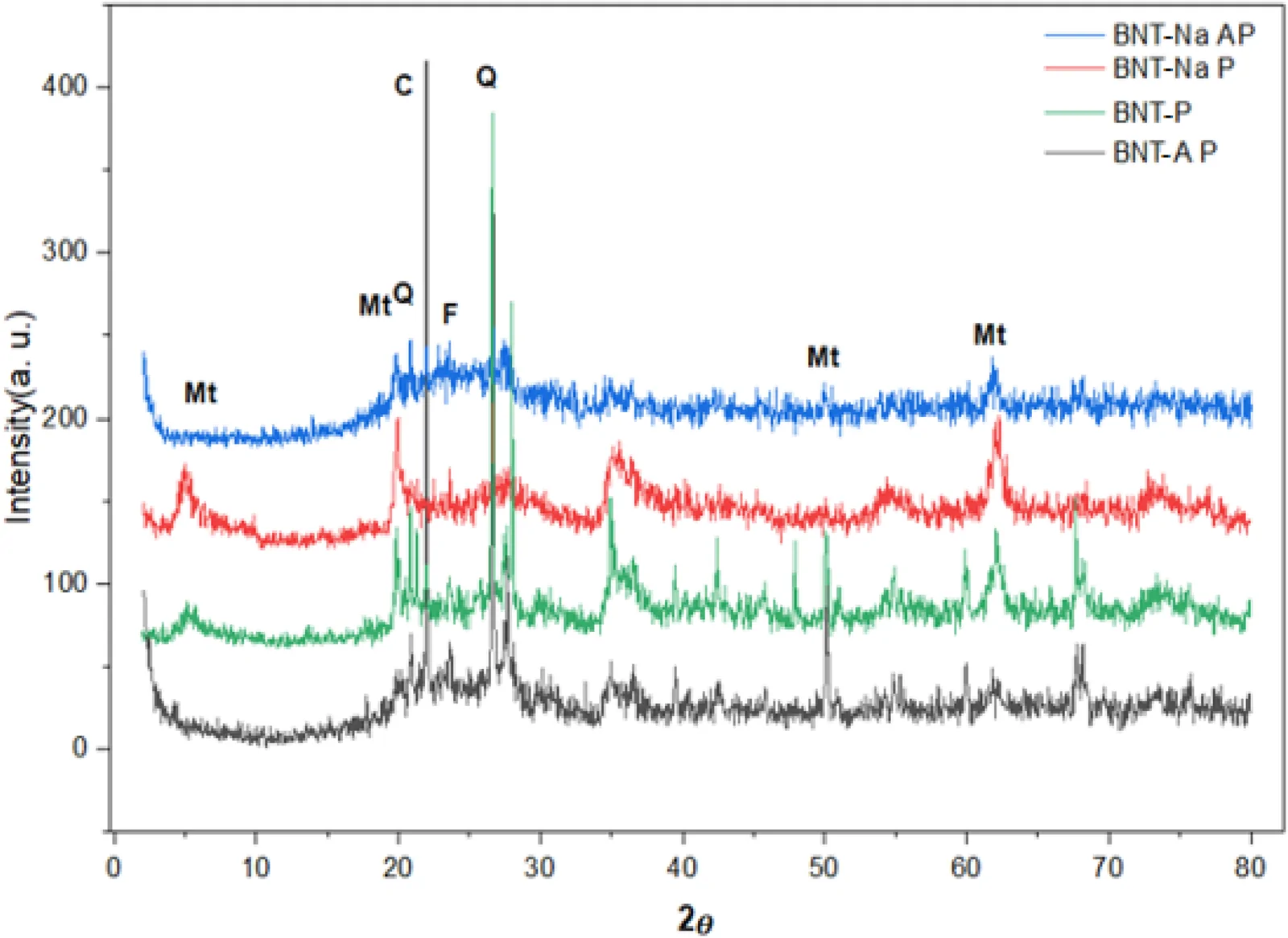
XRD patterns of bentonite samples after modification and calcination at 400 °C.

The broadening and weakening of the clay-related reflections further suggest a partial decrease in crystallinity. In contrast, the sharp reflection at approximately 2*θ* ≈ 26.6°, assigned to quartz, remains clearly visible, indicating the relative thermal stability of this accessory mineral phase under the applied calcination conditions. Overall, calcination at 400 °C modifies the structural order of the bentonite without producing clearly detectable new crystalline phases.^56^

### 3.3. Morphological and microstructural characterization by TEM

The TEM micrographs show the morphological differences between BNT-Na, BNT-NaAP and Co/BNT-NaAP (Fig. 5). BNT-Na shows overlapping plate-like particles aggregated densely. The darker central zones and more translucent edges are consistent with differences in local thickness due to piling up of clay layers.

**Fig. 5:**
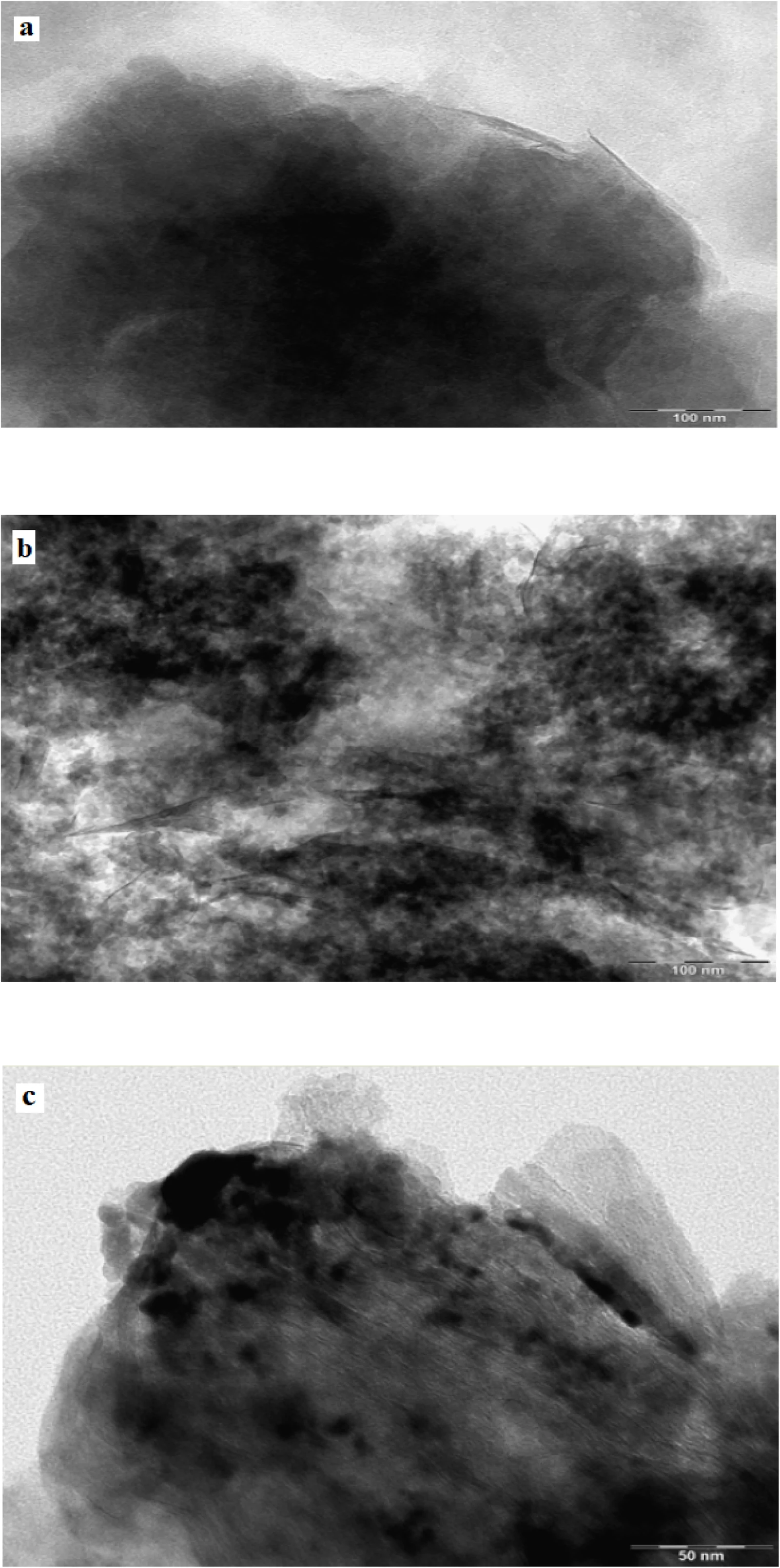
TEM micrographs of (a) BNT-Na, (b) BNT-NaAP, and (c) Co/BNT-NaAP.

The modified BNT-NaAP sample shows a heterogeneous texture with irregular aggregates and contrasting domains, which is consistent with changes in the particle organization.

In the Co/BNT-NaAP micrograph, several dark domains are dispersed over the lighter clay support, with some localised clustering. These domains may be related to particles containing cobalt, although thickness variations and diffraction contrast could also be responsible for their appearance.

### 3.4. Thermal behavior studied by TGA and DTA

Fig. S2 presents the TGA–DTA profiles of BNT-NaAP, which show a strong thermal event at low temperature (∼64 °C) and a broad feature centred at 254 °C. The low temperature event takes place in the region usually associated with the removal of physically adsorbed water from bentonite.^57^ However, the apparent exothermic direction of this figure does not allow unambiguous assignment to dehydration.

The large endotherm detected at approximately 254 °C could be associated with the gradual loss of more tightly bound water, possibly involving water associated with the Al-containing species added during modification. This is a tentative assignment. The annotation at 292 °C is not unambiguously identifying a separate resolved peak. Dehydroxylation and dehydration at different temperature ranges are exemplified by Al-pillared bentonite, where Cao *et al.*^58^ observed dehydration below about 231 °C and pillar dehydroxylation at about 497 °C.

Further mass changes at higher temperature may be due to structural dehydroxylation. Wolters and Emmerich^59^ observed that the dehydroxylation temperature of smectite depends on the octahedral structure and is most often observed in the range of 500–550 °C or 650–700 °C. Above about 600 °C the red curve levels off. This would suggest declining mass changes under the measurement conditions if it is a cumulative fractional mass loss.

### 3.5. BET surface area analysis

The N_2_ adsorption–desorption isotherms of the samples (see Fig. 6) are characteristic of type IV isotherms with H4-type hysteresis loops, as classified by IUPAC.

**Fig. 6:**
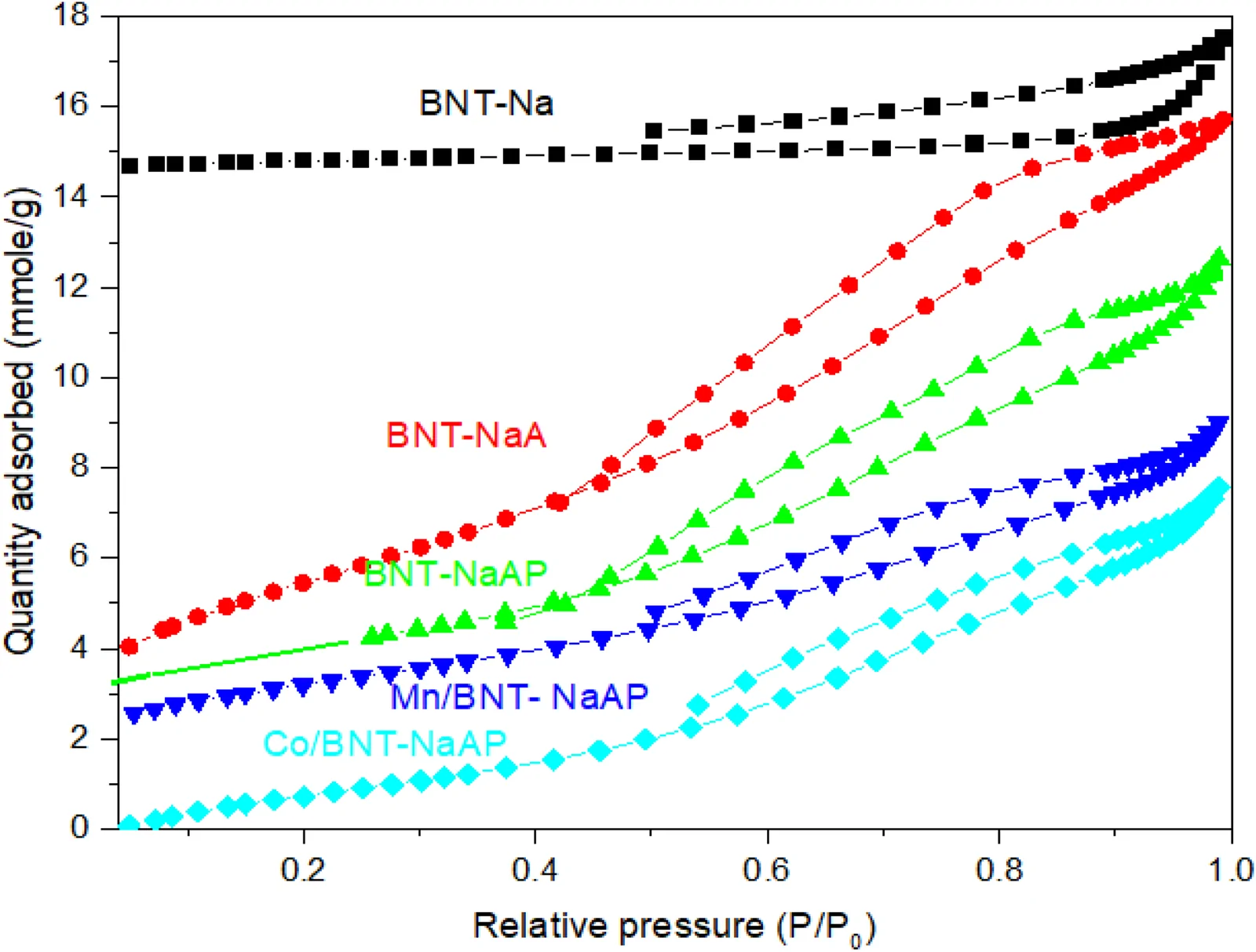
N_2_ adsorption–desorption isotherms of BNT-Na, BNT-NaA, BNT-NaAP, Mn/BNT-NaAP, and Co/BNT-NaAP samples.

Fig. 6 presents the BET linear plots for the studied samples in the relative pressure range of 0.05 to 0.30, corresponding to the valid application domain of the Brunauer–Emmett–Teller (BET) equation.

The BET plots were used to determine the monolayer adsorption capacities (*N*_m_) and specific surface areas (*S*_BET_) of the investigated bentonite-based samples.

As shown in Table 1, BNT-NaA exhibits the highest monolayer adsorption capacity (*N*_m_ = 4.497 mmol g^−1^), accompanied by a marked increase in specific surface area (*S*_BET_ = 438.84 m^2^ g^−1^). This substantial enhancement in textural properties is mainly associated with the structural modifications induced by sulfuric acid activation, including the formation of additional accessible pore space.^60^

**Table 1:** Monolayer adsorption capacity and BET surface area of the investigated materials

Sample	*N*_m_ (mmol g^−1^)	*S*_BET_ (m^2^ g^−1^)
BNT	0.472	46.26
BNT-Na	0.618	60.30
BNT-NaA	4.497	438.84
BNT-NaAP	2.160	210.42
Mn/BNT-NaAP	1.926	187.92
Co/BNT-NaAP	2.252	219.75

Moreover, the high monolayer adsorption capacity of the modified bentonite samples, especially BNT-NaA (*N*_m_ = 4.497 mmol g^−1^), agrees with the formation of a larger accessible surface area and enhanced pore accessibility. The increase in specific surface area and pore volume suggests that more adsorption sites are available after modification treatments. These textural changes may be associated with modifications of the interlayer environment and partial alteration of the clay structure during the modification processes.^61^

The BJH pore size distribution curves (see Fig. 7) reveal that the studied samples are mainly of mesoporous nature and the dominant pore populations are in the mesoporous range (2–50 nm). The distributions are mainly concentrated at relatively small pore diameters, followed by a progressive decrease in pore contribution with increasing pore diameter. BNT-NaA shows a relatively broader distribution and extends to larger pore diameters. The distribution of Mn/BNT-NaAP and Co/BNT-NaAP samples is mainly at smaller pore diameters. These differences indicate that the modification treatments alter the pore structure and accessibility of the bentonite-based materials. In particular, the incorporation of Mn- and Co-containing species appears to modify the pore-size distribution, although the BJH results alone do not allow the specific mechanism responsible for these changes to be established.^62^

**Fig. 7:**
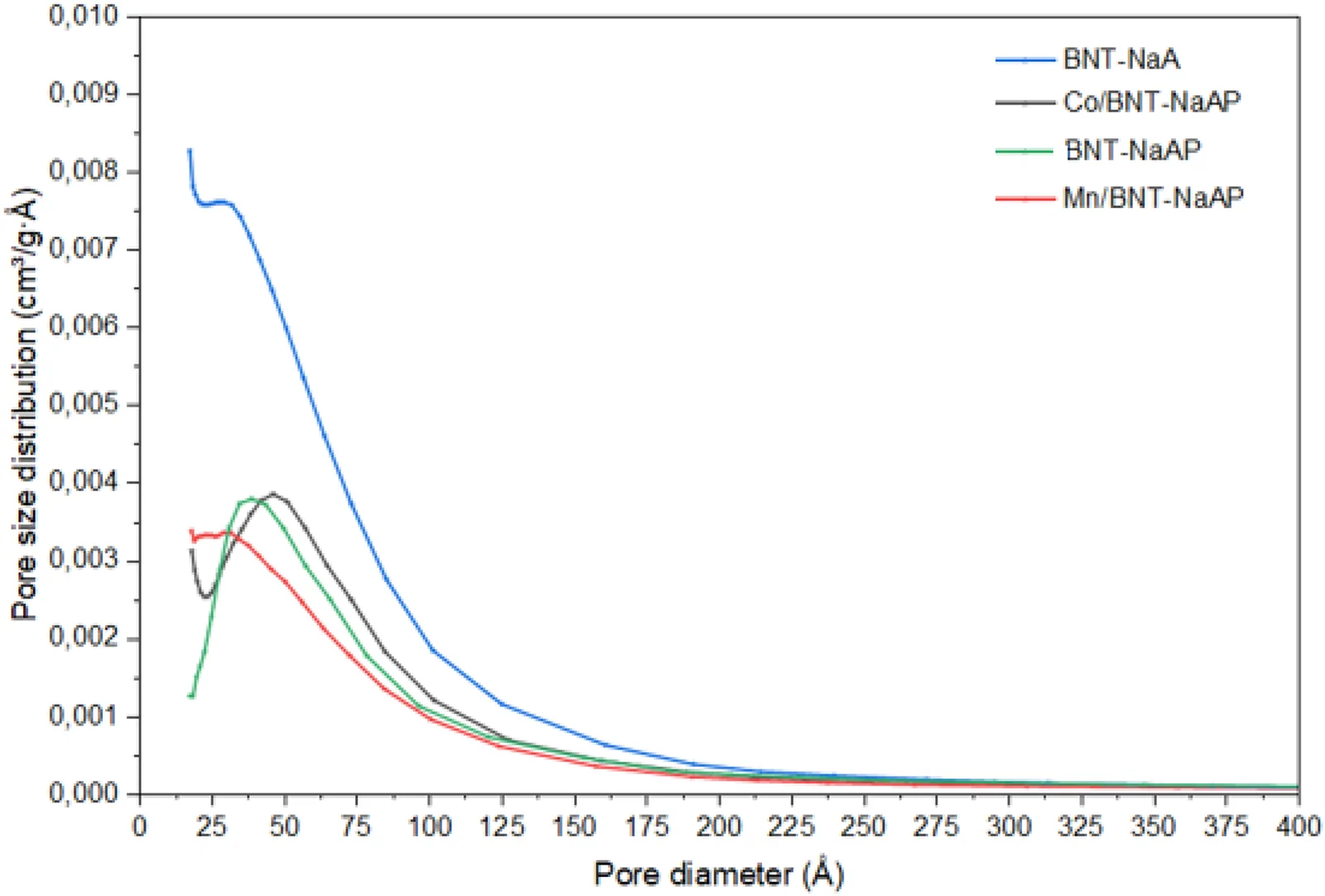
Pore-size distribution curves for adsorption of Co/BNT-NaAP, Mn/BNT-NaAP, BNT-NaA and BNT-NaAP samples.

The external surface area, *a*(*t*), was determined using the De Boer *t*-plot method by plotting the adsorbed amount of nitrogen (*N*_ads_, in mmol g^−1^) against the statistical thickness of the adsorbed layer (*t*, in nm) within the relative pressure range of 0.35 to 0.50. These plots are presented in Fig. S3.

Table S2 reveals a significant increase in the specific surface area *S*_BET_ from 60.3 m^2^ g^−1^ for BNT-Na to 438.8 m^2^ g^−1^ for BNT-NaA, which demonstrates the effectiveness of acid activation in improving mesoporosity thru cation leaching and partial delamination of the layers.

BNT-NaAP exhibits a specific surface area (*S*_BET_) of 210.42 m^2^ g^−1^, representing approximately a threefold increase compared to BNT-Na. The textural properties of BNT-NaAP are in accordance with a mesoporous pore structure. The relatively high surface area and pore volume of BNT-NaAP could be related to the combined effects of acid-induced structural modification and subsequent treatment with Al_13_ species.^63^ However, because a well-defined *d*_001_ reflection is not observed for BNT-NaAP, the formation of a well-ordered pillared structure cannot be confirmed by XRD alone.

The relatively high surface areas observed for these materials are therefore more appropriately associated with the structural modifications and pore development induced by acid activation and subsequent treatment rather than with a clearly demonstrated ordered pillared structure.

The external surface areas calculated using the t-plot method are generally lower than those obtained by the BET method for most samples. The highest total pore volume (0.54 cm^3^ g^−1^) was observed for BNT-NaA, which signifies an increase in pore volume after acid-activation treatment and enhanced pore accessibility. This relatively high pore volume may be due to acid-induced structural changes such as partial leaching of structural cations and changes in the interlayer and pore structure.^64^

The possible contribution of the Al_13_ treatment to the textural properties can be discussed in the light of previous studies on Al-pillared clays. Zhao *et al.* prepared Al-pillared mesoporous montmorillonite using concentrated Al_13_ solution and observed an enhancement in specific surface area and a more uniform pore-size distribution.^65^

The specific surface area of Mn/BNT-NaAP and Co/BNT-NaAP catalyst is 187.92 and 219.75 m^2^ g^−1^, respectively. Although these values are lower than BNT-NaA (438.84 m^2^ g^−1^), both materials still have relatively high surface areas.

The higher *S*_BET_ observed for Co/BNT-NaAP compared with Mn/BNT-NaAP may reflect differences in the interaction of the respective metal precursor species with the clay support. However, the specific mechanism cannot be established from BET data alone.^66^

The differences in surface area after metal impregnation can be also compared with previous results for cobalt loaded clay supports. Al-pillared clays containing various Co contents were studied by Macías-Quiroga *et al.*, who reported variations in their textural properties depending upon the loading.^67^ Their results provide context for discussion of the effect of cobalt incorporation on porosity.

The BJH pore size distribution analysis shows that all investigated samples are mainly mesoporous, with pore diameters mostly in the mesoporous range as defined by IUPAC (2–50 nm).^68^ The average pore diameters were about 4.80, 4.91, 5.13 and 5.27 nm for BNT-NaA, BNT-NaAP, Mn/BNT-NaAP and Co/BNT-NaAP, respectively. BNT-NaA had the smallest mean pore diameter and BNT-NaAP, Mn/BNT-NaAP and Co/BNT-NaAP had slightly higher values. This progressive change indicates that the consecutive modification treatments affect the pore-size properties of the bentonite-based materials.

Such variations may reflect changes in pore structure and accessibility induced by the successive treatments.^69–71^ However, BJH analysis alone does not allow the specific mechanisms responsible for the observed changes in pore-size distribution to be established.

In addition, previous studies have demonstrated the potential of chemically modified and pillared clays as catalyst supports. Liu *et al.*^72^ found that acid activation and subsequent pillaring of bentonite significantly altered the textural and structural properties of the bentonite, leading to enhanced catalytic performance of the supported catalyst. These results underline the importance of the surface area, the pore structure and the structural modification of the clay-based catalytic supports.

### 3.6. Fractal analysis

The adsorption branches of the isotherms of BNT-Na, BNT-NaA, BNT-NaAP, Co/BNT-NaAP and Mn/BNT-NaAP were employed for fractal dimension analysis. As illustrated in Fig. S4, the FHH (Frenkel–Halsey–Hill) fractal model was applied, revealing linear correlations between 
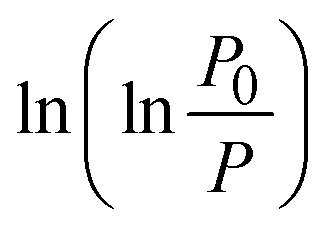
 and 
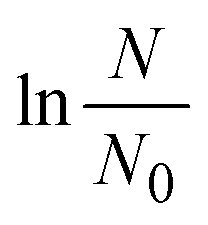
.

The fractal dimension (*D*) values calculated for the series of BNT-based samples are 2.61, 2.66, 2.95, 2.88, 2.90, and 2.91, corresponding respectively to BNT, BNT-Na, BNT-NaA, BNT-NaAP, Mn/BNT-NaAP, and Co/BNT-NaAP. All these values are in the range of 2 to 3, which are indicative of fractal surfaces, where increasing *D* corresponds to increasing surface roughness and complexity.^73^

### 3.7. Reducibility of the supports and cobalt-containing materials

Fig. S5 shows the H_2_-TPR profiles of BNT, BNT-Na and BNT-NaAP. All three samples exhibit a broad feature centred around ∼500–750 °C superimposed on a rising background. The apparent maxima of BNT and BNT-Na are observed at around 650–700 °C whereas the main characteristic of BNT-NaAP is observed at lower temperature, about 600–650 °C. The broad profiles indicate overlapping contributions rather than a single, well resolved reduction process.

The sodium exchange causes moderate changes in the profile when compared to raw BNT, whereas the Al_13_ modification causes a more pronounced decrease of the signal in the main reduction region. This shows that the treatment alters the support response to conditions that are reducing.

The H_2_-TPR profile of Co/BNT-NaAP presents a broad feature at about 600 °C and a more defined maximum at about 780 °C (Fig. S6). Such features imply reduction processes involving species with different chemical environments and resistance to reduction.

Supported cobalt oxides are typically reduced sequentially from Co_3_O_4_ to CoO and then to metallic Co.^74^ However, the two observed maxima cannot be assigned to these individual transformations directly without determining the initial cobalt phases and evaluating the contribution of the unloaded support.

The high-temperature feature may reflect cobalt species strongly interacting with the aluminosilicate support, which would hinder their reduction. Such an interpretation is consistent with observations reported for cobalt interacting with silica-based supports.^75^

### 3.8. Catalytic performance in soot oxidation

The curve Co/BNT-NaAP attains its maximum at about 520 °C, compared with 605 °C for BNT-NaAP and 680 °C for soot alone (see Fig. 8). These shifts would point to facilitated soot oxidation; the largest temperature reduction being associated with Co/BNT-NaAP.

**Fig. 8:**
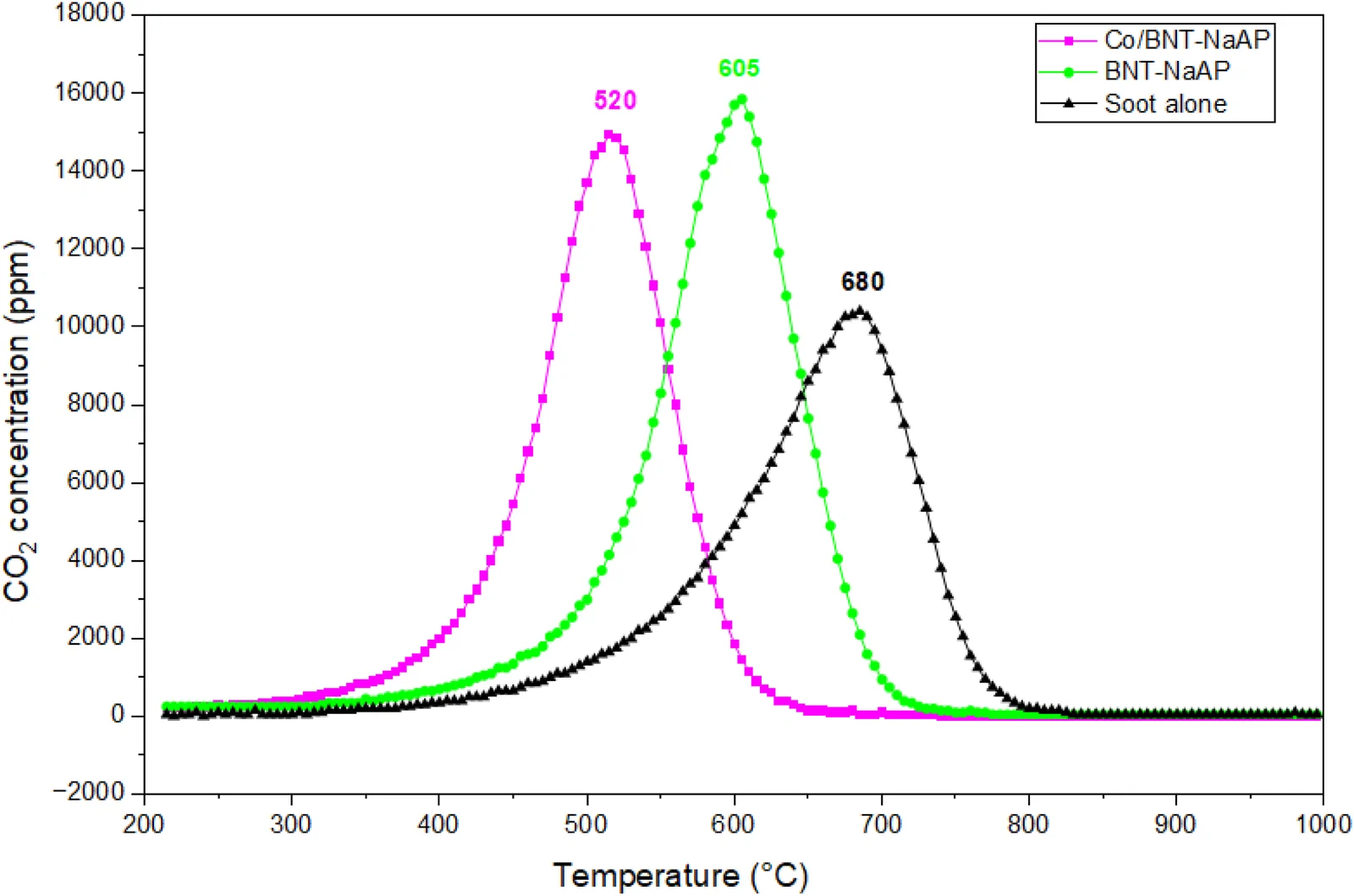
CO_2_ evolution during soot oxidation under O_2_.

Fig. 9 presents the soot conversion profiles as a function of temperature for Co/BNT-NaAP, BNT-NaAP, and soot alone.

**Fig. 9:**
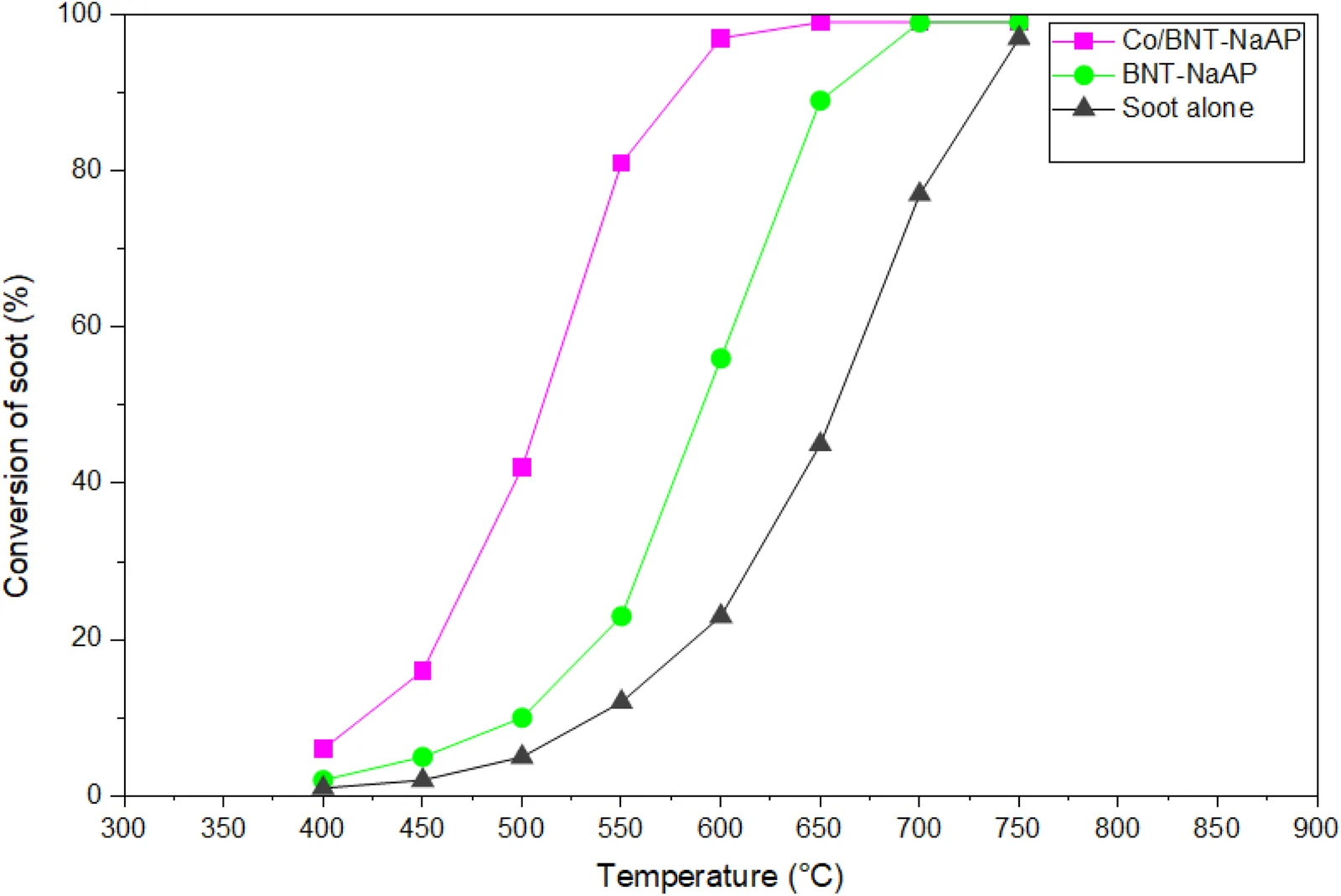
Soot conversion as a function of temperature over Co/BNT-NaAP and BNT-NaAP compared with soot alone.

As shown in Table 2, Co/BNT-NaAP has the lowest *T*_10_, *T*_50_, and *T*_90_ values, suggesting that soot oxidation occurs at lower temperatures than for BNT-NaAP and soot alone. The estimated *T*_50_ is of the order of 510 °C, compared to 590 °C for BNT-NaAP and 660 °C for soot only. These correspond to reductions of approximately 80 and 150 °C, respectively. BNT-NaAP shows an intermediate behaviour, while soot alone requires the highest temperatures over the whole conversion range. The order of the apparent activity is thus Co/BNT-NaAP > BNT-NaAP > soot alone.

**Table 2:** *T*_10_, *T*_50_, and *T*_90_ for soot oxidation under O_2_

Sample	*T*_10_ (°C)	*T*_50_ (°C)	*T*_90_ (°C)
Soot alone	534	660	730
BNT-NaAP	500	590	650
Co/BNT-NaAP	420	510	580

Table 3 shows a comparison of soot oxidation performance of Co/BNT-NaAP with some selected cobalt-containing catalysts reported in the literature. The *T*_50_ of Co/BNT-NaAP was reported to be ∼510 °C, which is 10, 29 and 40 °C lower than that of CuCo_2_O_4_-N, CuCo_2_O_4_-A and CuCo_2_O_4_-C, respectively.^76^ In contrast, MnCo_2_O_4_ exhibited a lower *T*_50_ of 446 °C in O_2_/He atmosphere without NO.^77^ The values place Co/BNT-NaAP in the temperature regime reported for the selected cobalt containing catalysts.

**Table 3:** Comparison of the soot oxidation performance of Co/BNT-NaAP with selected cobalt-based catalysts reported in the literature

Catalyst	Reaction atmosphere	*T*_50_ (°C)	*T*_m_ (°C)	Reference
Co/BNT-NaAP	O_2_-containing atmosphere	510	520	This work
CuCo_2_O_4_-N	10 vol% O_2_	520	—	76
CuCo_2_O_4_-A	10 vol% O_2_	539	—	76
CuCo_2_O_4_-C	10 vol% O_2_	550	—	76
MnCo_2_O_4_	O_2_/He without NO	446	—	77
Co_90_Ce_10_-CC clay-supported	18% O_2_ + 0.1% NO/He	—	440	78

The CO_2_ evolution peak temperature of Co/BNT-NaAP was about 520 °C, compared to 440 °C for clay-supported Co_90_Ce_10_-CC.^78^ However, the former was checked in NO containing atmosphere and the present experiments were carried out without addition of NO. This restricts direct comparison because of differences in oxygen concentration, heating rate, catalyst to soot ratio and conversion determination. In particular, in this work the conversion values were obtained from CO_2_ measurements only.

## 4. Conclusion

In this study, a series of bentonite-based materials were prepared through sodium exchange, sulfuric acid activation, Al_13_ polycation treatment, and subsequent Co and Mn impregnation. The combined FTIR, XRD, TEM, nitrogen adsorption–desorption BET, and fractal analyses demonstrated that the sequence of modification treatments strongly influenced the structural and textural properties of the bentonite.

Sulfuric acid activation produced a pronounced modification of the clay structure and significantly enhanced its textural properties. In particular, BNT-NaA exhibited the highest specific surface area, reaching 438.84 m^2^ g^−1^, together with a total pore volume of 0.54 cm^3^ g^−1^, indicating the development of a well-developed porous network. These changes are primarily connected with the leaching of structural cations and the partial destruction of the original layered structure during the acid treatment.

The XRD analysis showed that the interlayer region of BNT-P and BNT-NaP was expanded after treatment with Al_13_ polycations and the *d*_001_ spacings were 16.96 and 18.03 Å, respectively. The results are indicative of incorporation of Al_13_ species between the layers in the raw and the sodium exchanged samples of bentonite. However, the fact that no distinct *d*_001_ reflection is observed for BNT-AP and BNT-NaAP suggests that the strong acid treatment caused a significant breakdown of the original lamellar structure and that the subsequent Al_13_ treatment was not able to reconstruct a sufficiently ordered layered framework. Thus, based on XRD alone, the formation of a well-ordered Al_13_-pillared structure cannot be confirmed for these acid-activated materials.

Despite the observed structural disorder, BNT-NaAP still showed a relatively high specific surface area of 210.42 m^2^ g^−1^ and exhibited considerable mesoporous characteristics. The Co/BNT-NaAP and Mn/BNT-NaAP still maintained relatively high specific surface areas of 219.75 and 187.92 m^2^ g^−1^, respectively, after metal impregnation. The fractal analysis revealed that the modification treatments led to an increase of the surface roughness and heterogeneity with fractal dimensions varying from 2.61 to 2.95.

Overall, these results emphasize the significant effect of the modification sequence on the structural and textural properties of bentonite. The resulting materials, particularly those exhibiting high surface area and developed mesoporosity, may serve as promising supports for further catalytic investigations.

The soot oxidation tests in O_2_ showed that the conversion temperatures of Co/BNT-NaAP were lower than those of BNT-NaAP and soot alone. These results demonstrate that additive of cobalt increased catalytic activity under the investigated conditions and confirm the potential application of modified bentonite as catalyst support for soot oxidation.

## Conflicts of interest

There are no conflicts to declare.

## Supplementary Material

RA-OLF-D6RA07334D-s001

## Data Availability

All data supporting the findings of this study are included within the manuscript. Supplementary information (SI) is available. See DOI: https://doi.org/10.1039/d6ra07334d.
