# Supplementary material for: Synthesis of Keggin-Al13-modified bentonite materials with enhanced mesoporosity: structural and textural characterization and application in soot oxidation
Source: RSC Adv. 2026 Nov 10. Online ahead of print. doi: 10.1039/d6ra07334d (PMC13749080; doi:10.1039/d6ra07334d)
Supplement: Supplementary file 1 [file RA-OLF-D6RA07334D-s001.pdf]

This journal is © The Royal Society of Chemistry

**Table S1.** Major oxide composition of natural bentonite clay (wt.%)

| Main oxides | $SiO_2$ | $Al_2O_3$ | $Fe_2O_3$ | MgO | CaO | $Na_2O$ | $K_2O$ | $TiO_2$ | MnO | L.O. I |
|---|---|---|---|---|---|---|---|---|---|---|
| Weight percent (wt.%) | 69.4 | 14.7 | 1.2 | 1.1 | 0.3 | 0.5 | 0.8 | 0.2 | 0.05 | 11 |

L. O. I = Loss on ignition at 900 °C

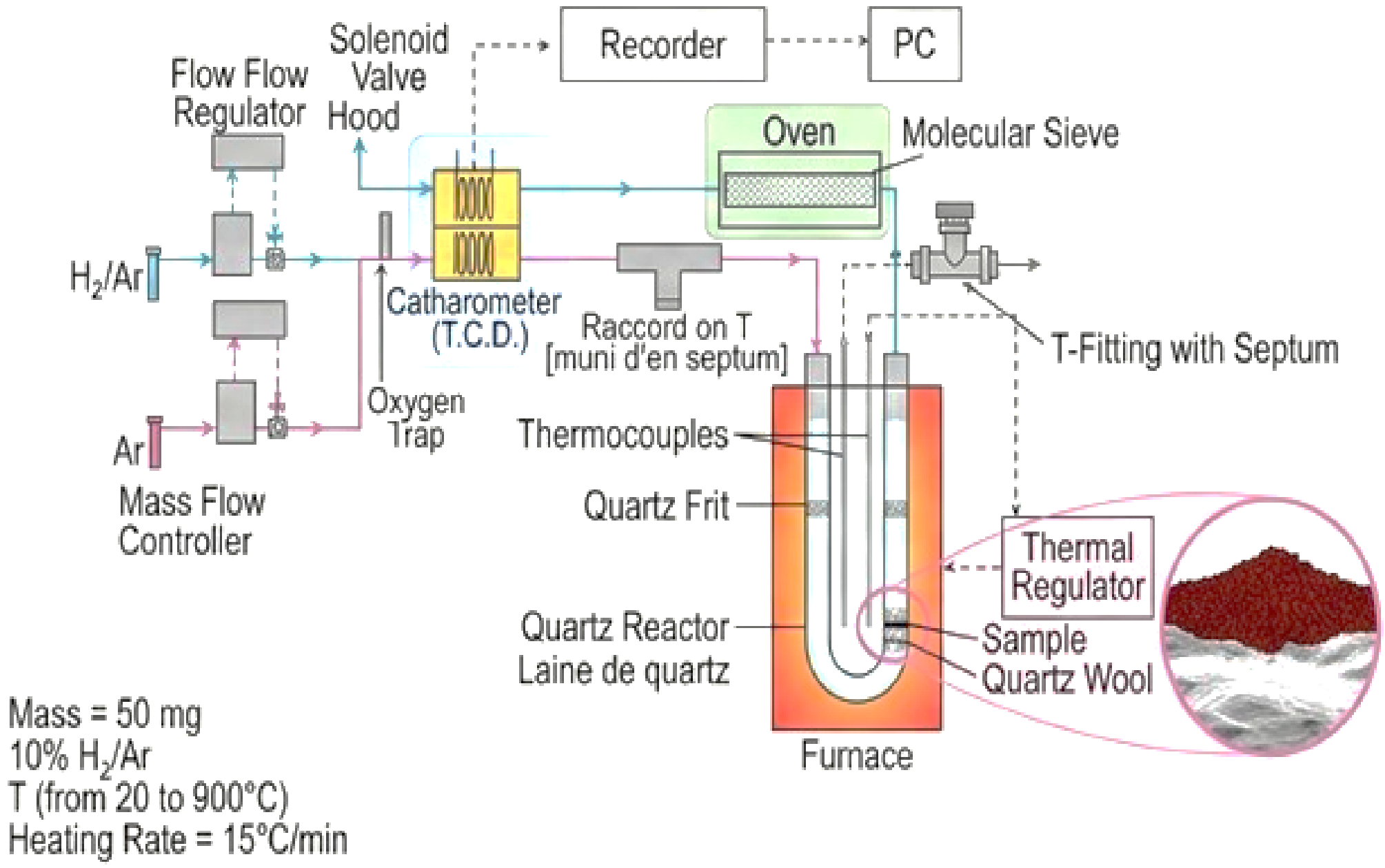


**Figure S1.** Schematic diagram of the experimental setup for Temperature-Programmed Reduction (TPR) analysis.

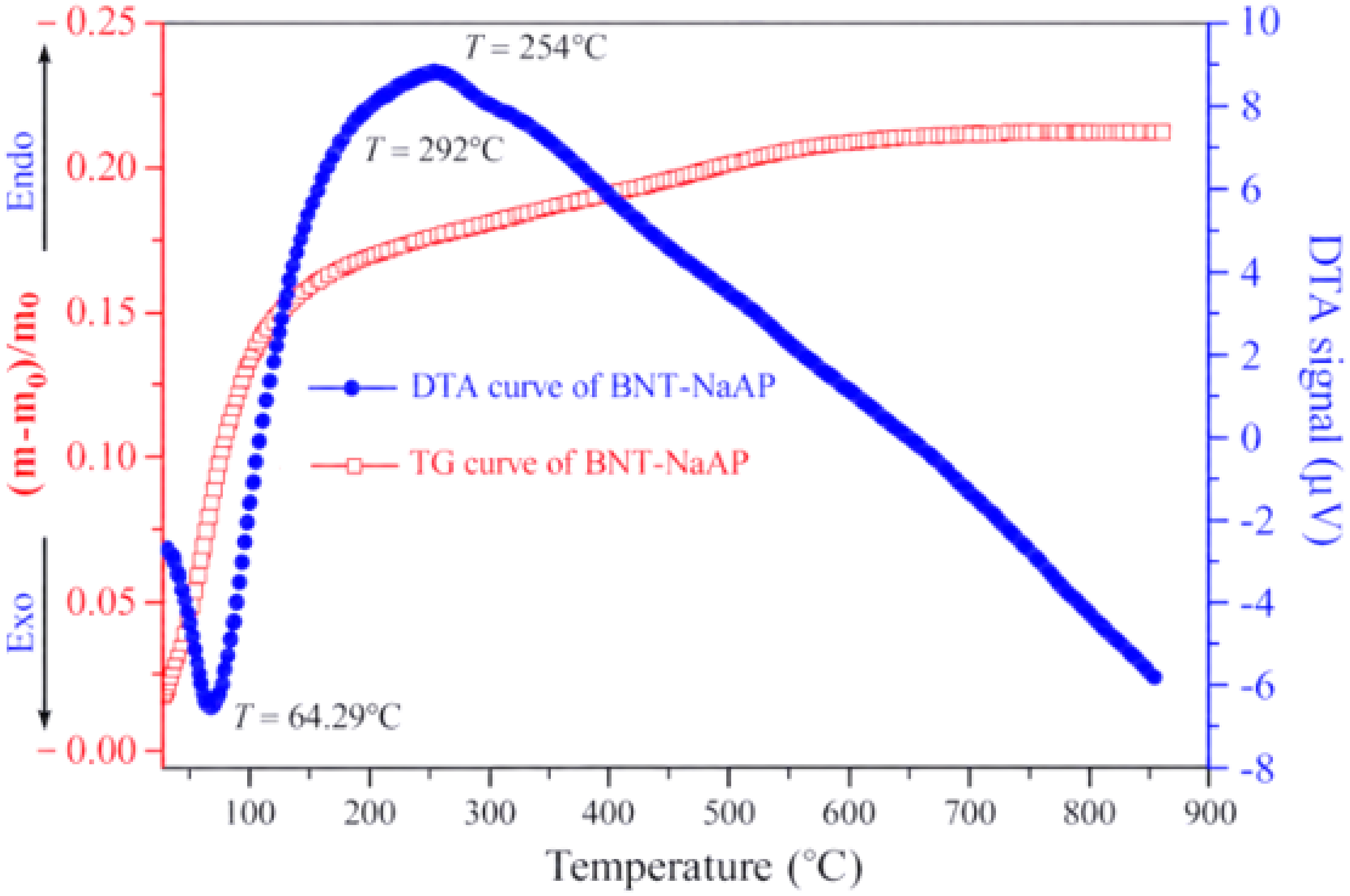


**Figure S2**. TGA and DTA curves of BNT-NaAP.

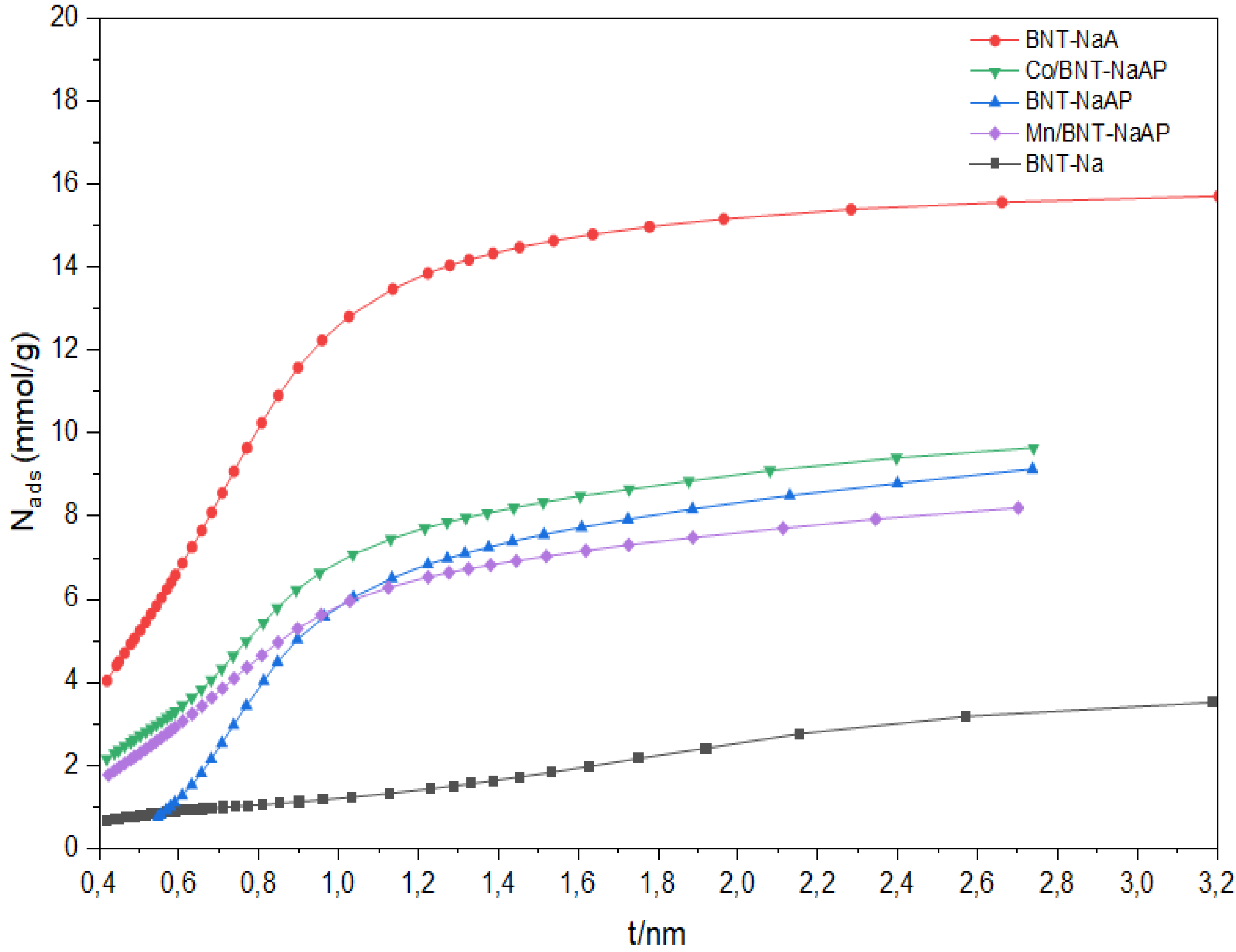


**Figure S3.** Determination of external surface area of studied samples using the De Boer t-plot method.

**Table S2.** Textural measurement results of the studied samples.

| Sample | $S_{ext}$ ($m^2/g$) | $V_P$ ($cm^3/g$) | Average pore diameter (nm) | Mean pore diameter from BET (nm) | BJH pore diameter (Å) | Total pore volume ($cm^3/g$) |
|---|---|---|---|---|---|---|
| BNT | 37.42 | 0.0021 | 0.97 | — | — | — |
| BNT-Na | 24.86 | 0.0344 | 3.88 | — | — | — |
| BNT-NaA | 13.28 | 0.0330 | 4.80 | 4.942 | 48.11/54.89 | 0.54 |

| | | | | | | |
|---|---|---|---|---|---|---|
| **BNT-NaAP** | 38.71 | 0.2108 | 4.91 | 5.770 | 52.35/63.31 | 0.31 |
| **Mn/BNT-NaAP** | 30.43 | 0.2022 | 5.13 | 6.022 | 60.43/62.37 | 0.33 |
| **Co/BNT-NaAP** | 31.91 | 0.2475 | 5.27 | 5.827 | 56.19/60.28 | 0.28 |

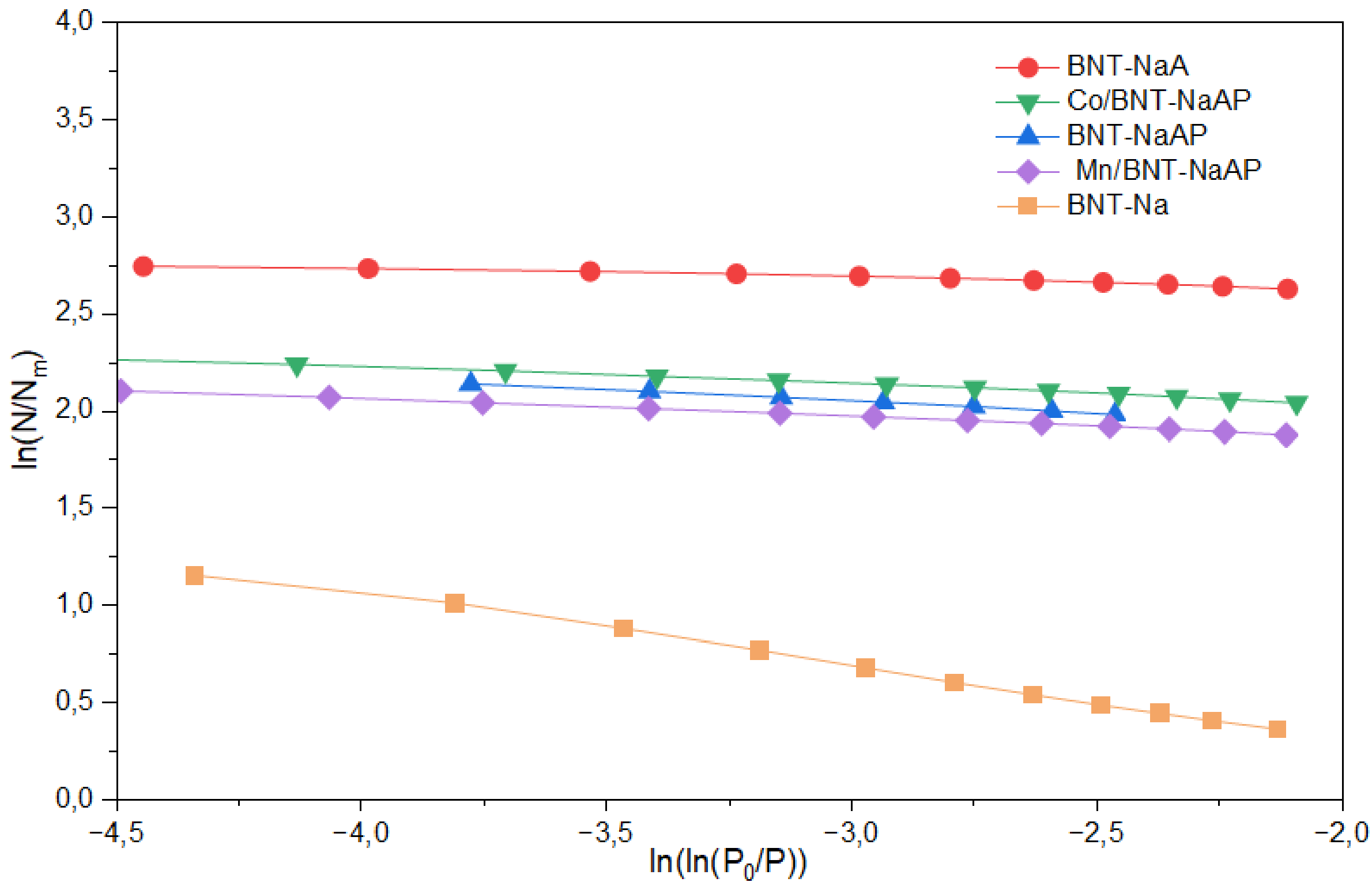


**Figure S4.** Fractal dimension analysis of the studied samples based on the FHH model.

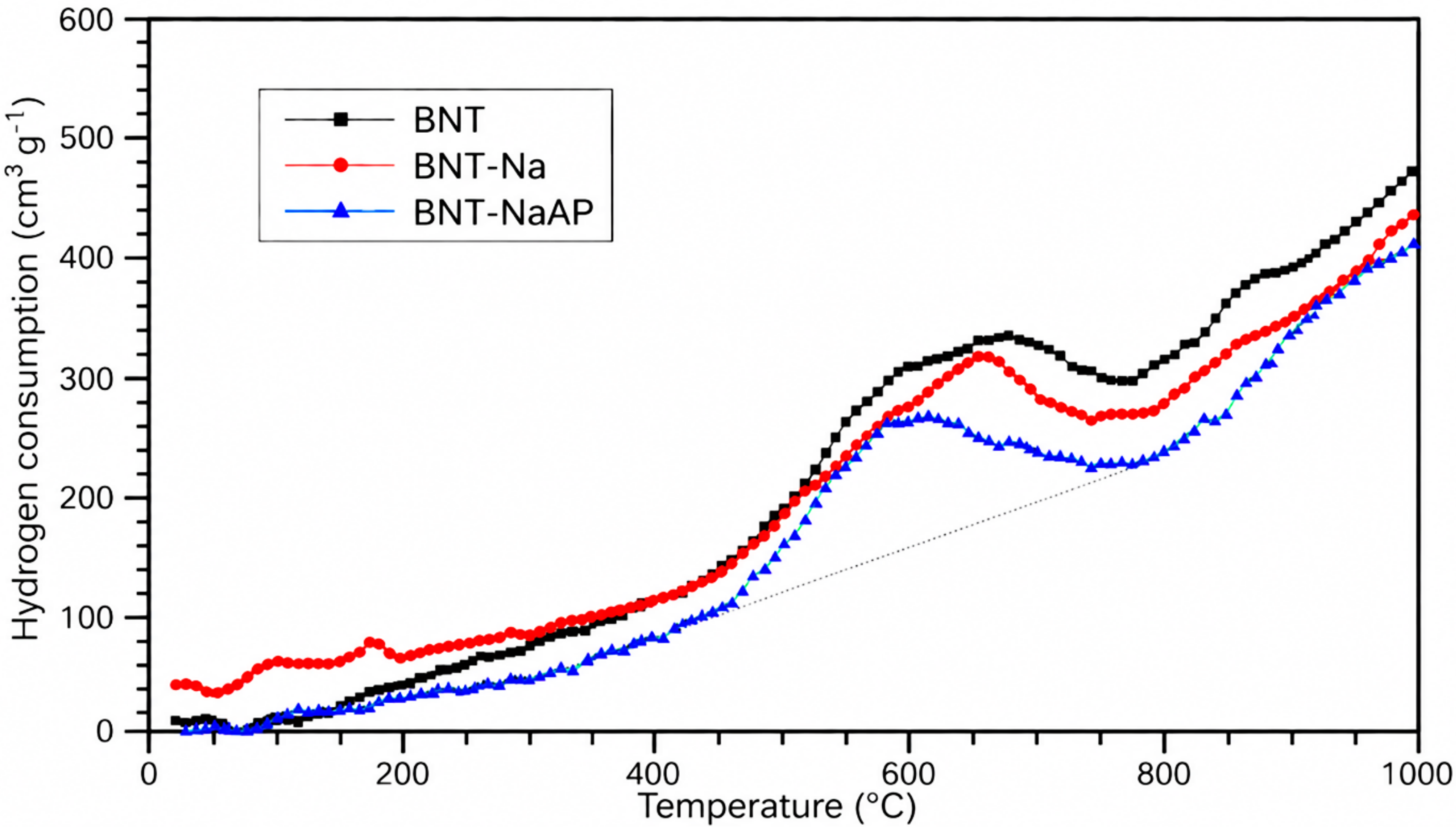


**Figure S5.** $H_2$-TPR profiles of BNT, BNT-Na and BNT-NaAP supports.

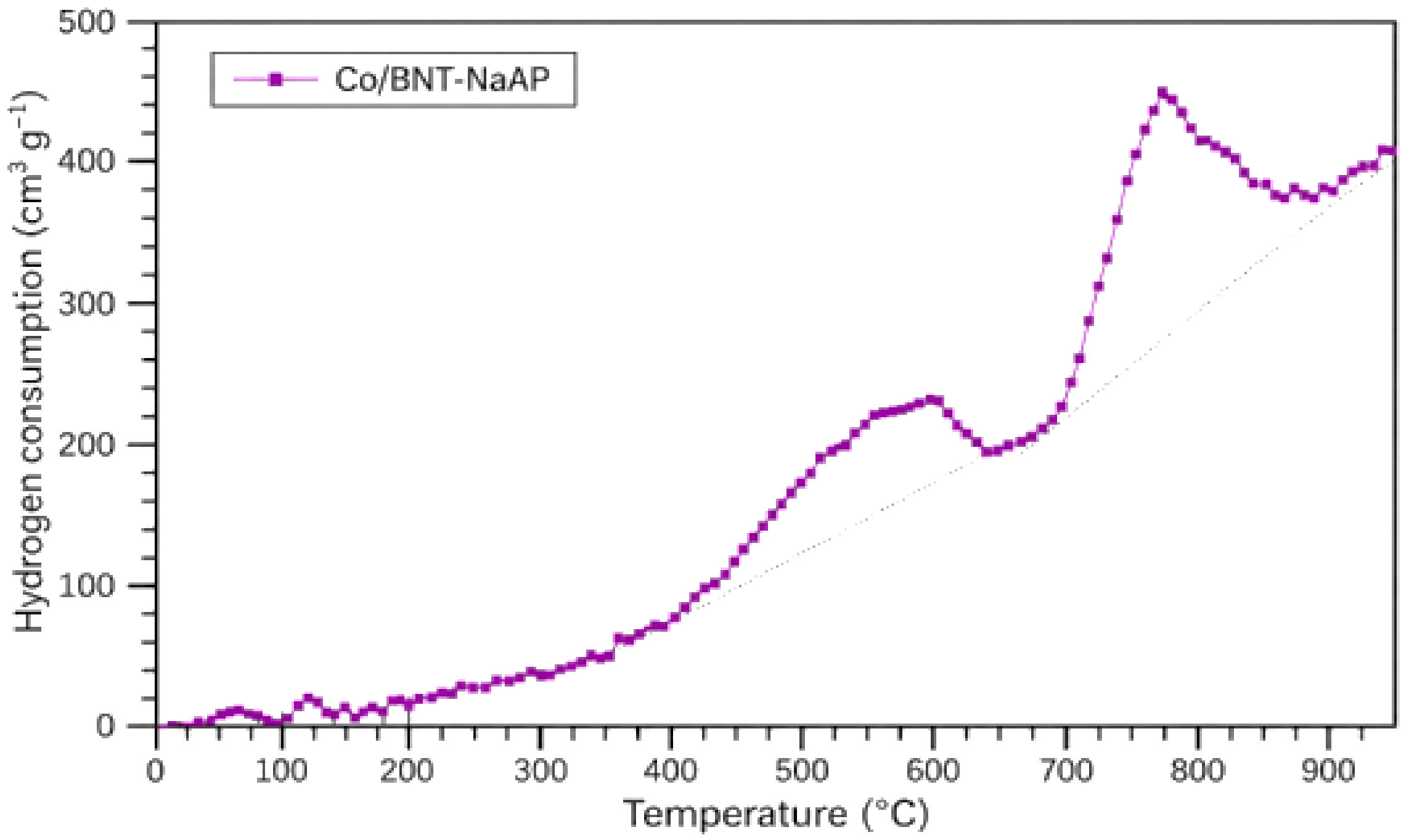


**Figure S6.** $H_2$-TPR profile of the Co/BNT-NaAP catalyst.
